# Awareness and Need for Knowledge of Health and Safety among Dairy Farmers Interviewed in Uganda

**DOI:** 10.3389/fpubh.2016.00137

**Published:** 2016-06-28

**Authors:** Christina Lunner-Kolstrup, Tonny Kiggundu Ssali

**Affiliations:** ^1^Department of Work Science, Business Economics and Environmental Psychology (AEM), Swedish University of Agricultural Sciences (SLU), Alnarp, Sweden; ^2^Faculty of Agriculture, Uganda Martyrs University (UMU), Kampala, Uganda

**Keywords:** dairy farmers, physically demanding, risk factors, agrochemicals, injuries, developing countries, Uganda

## Abstract

**Introduction:**

Safe working conditions are essential for healthy living and for ensuring food security among farmers and farm communities in developing countries. There is limited research on this topic, and documentation is essential to understand and change patterns of human health and safety.

**Methods:**

In May 2014, six male and female farmers on four dairy farms in Uganda and a female veterinarian were interviewed about their awareness and attitudes to agricultural risk factors, health, and safety. In addition, transect walks were conducted on the four dairy farms.

**Results:**

The dairy farmers reported health and safety concerns, e.g., diarrhea, coughs, fever, cuts while using machetes in plantations, bruises when handling animals, and dizziness and poisoning symptoms from using different agrochemicals, and considered these an occupational hazard. The most important topic mentioned was the use of agrochemicals and drugs on livestock. The farmers spray their animals with insecticides to prevent ticks, lice, tsetse flies, and other biting nuisance flies, using a backpack or hand sprayer. Spraying is conducted without personal protection equipment, which is considered too expensive and difficult to obtain. The farmers reported that they usually feel dizzy, vomit, and have pain and a burning feeling in their face and eyes after spraying. The symptoms are sometimes so severe that they require treatment. In such cases, the farmers buy medication without a prescription at the local drugstore, where the storekeeper often has limited or no knowledge of agrochemicals or drugs except for dosage. Agricultural health and safety training in the region is non-existent, and the farmers expressed a need and desire for improvements in this area.

**Conclusion:**

The level of knowledge and awareness of agricultural health and safety risks, disease, and injury prevention among the Ugandan dairy farmers interviewed was low. The farmers mentioned few agriculture-related complaints, injuries, or diseases except poisoning from using agrochemicals. Training on health and safety in Ugandan agriculture is urgently needed.

## Introduction

Agriculture engages about 1.3 billion people worldwide, almost 60% of whom live in developing countries (1–5). The agriculture sector comprises different branches, such as crop, horticultural, and livestock production, which involve a range of work tasks resulting in agricultural workers being exposed to a diverse array of occupational hazards (2, 6). Agriculture has been identified as one of the most hazardous sectors in the world, and it is estimated that of 335,000 fatal work-related accidents occurring worldwide every year, some 170,000 involve agricultural workers (4). Large numbers of the world’s agricultural workers also suffer serious work-related injuries and diseases caused by machinery, chemicals, and animals (4).

Although agricultural farms in many developed countries are highly mechanized, operate on a large scale and tend to practice monoculture, farming in many developing countries is much more labor-intensive, non-mechanized, and integrates both crop and livestock production. These differences have a significant bearing on the levels of risk awareness and attitudes to preventing injuries and diseases within the sector (3–5).

It is well known that dairy farming is associated with demanding and hazardous risk factors, such as difficult working postures and movements and repetitive and monotonous work tasks giving rise to musculoskeletal disorders. It is also associated with exposure to noise, vibration, dust, weather, pesticides, zoonotic diseases (diseases and infections that are naturally transmitted between vertebrate animals and humans), excessively long hours, and handling of livestock, which can affect the health and safety of farmers and farm workers (2, 5, 7–15). Additional factors identified as contributing to injuries and ill-health among the farm population are fatigue, time pressure, stress, poor equipment maintenance, lack of personal protection equipment (PPE), poor knowledge and awareness, and human error (16, 17).

Many of these hazards and health and safety issues related to dairy farming are more or less similar worldwide, but vary depending on production system, socioeconomic context, and conditions (6, 16, 18–20). Systematic studies on agriculture-related health and safety in developing countries are scarce (21). The few studies available specifically addressing occupational health and safety issues among farm populations in Africa show high incidences of self-reported acute and chronic injuries (22–24), comprehensive exposure to agrochemicals, a high incidence of poisoning (25–27), and a high incidence of infectious diseases relating to agriculture (28). Very few studies have examined the health and safety of farmers and their families in Uganda. Records from the 1980s reveal that the annual number of cases of pesticide poisoning in Uganda at that time was 272,000 (29). Kobusingye et al. (24) found that injuries among people living in rural areas gave rise to an annual mortality rate of 92 per 100,000 and that injury-related disabilities had a prevalence proportion of 0.7%.

The available research regarding the health and safety of farmers in developing countries is limited, and documentation is essential in order to understand and change behavioral patterns and attitudes in this regard.

The overall aim of this study was to increase knowledge and highlight agriculture-related human health and safety issues, which in future could lead to fewer injuries, illnesses, and other negative consequences for the livelihood of farmers and their families in developing countries. To achieve this aim, interviews were conducted with Ugandan dairy farmers and family members regarding their attitudes, how they perceived risk factors, health, and safety in an agricultural context, and how it affected their daily lives and livelihood at large. The study also focused on lifting existing needs, possibilities, and obstacles for future research regarding issues on agricultural health and safety among dairy farmers in Uganda.

## Materials and Methods

### Study Design and Ethical Aspects

The study comprised a qualitative, small-scale, cross-sectional study using semi-structured interviews and transect walks. The intention was not to generalize, but to explore and highlight Ugandan dairy farmers’ subjective knowledge and experiences, as well as needs, possibilities, and obstacles regarding agricultural health and safety and the effect on livelihood (30). For this purpose, inductive qualitative methodology was appropriate (31, 32). The motive for choosing a qualitative approach using interviews and transect walks was to create a more nuanced picture, gain a deeper understanding of the participants’ perceptions and experiences, and obtain comparable and reliable data, while, at the same time, keeping a fairly open framework to follow-up leads (33). These methods were chosen instead of a questionnaire, as farmers are difficult to reach by postal mail and illiteracy among farmers is common in the region. The participants were provided with oral information about the project and the purpose of the study, and anonymity and the voluntary nature of their participation were explained. No application was made to the Ethical Committee as the study was a pilot study, but current national guidelines based on the Helsinki Declaration concerning research ethics, anonymity, voluntariness, confidentiality, and retention of data were considered and fulfilled (34). The study was conducted during the period of May 19–23, 2014 (30).

### Study Participants

Agricultural statistics presented here on number of farming households (farms), farmers, animals, and herd sizes in Uganda are based on estimates provided by the Ministry of Agriculture, Animal Industry and Fisheries (35). According to the latest National Livestock Census (35, 36), the number of cattle and households owing cattle in 2008 was estimated to be 11.4 and 1.7 million, respectively. Households owing cattle represented 26% of all households in Uganda in 2008. More than 90% of Ugandan cattle farmers are smallholders with an average herd size of seven cattle per household and a milk production level of 8.5 l per cow and week (no information is available on dairy cow herd size) (35–37). The Western region has the highest density of dairy farmers and milked cows in the country (0.41 million milked cows, compared with 1.52 for the entire country) (36, 37). Therefore, study participants representative of an average Ugandan dairy farm were chosen from the cattle-intensive Western Uganda (specifically from Mbarara district, part of the Ankole sub-region). Important criteria for participation were that the farmers had a dairy production enterprise representative for Uganda and were willing to share experiences and knowledge about agricultural risks and health and safety issues. The study participants were a convenience sample selected by the Ugandan University colleague, an extension advisor, the corresponding author, and in accordance with the abovementioned criteria. The study ended up comprising four dairy farms, three male and one female dairy farmer, a female farm family member, a hired male dairy farm worker, and a female veterinarian who was also a university lecturer. A description of the farms and participants is provided in the Section “Results” of this paper.

### Interviews and Transect Walks

An interview guide was developed and tested by the research team (both authors) prior to the study and included questions about:

- Demographics of the dairy farms (e.g., size, ownership, subsistence farming or products for sale in the local market, workforce, type and number of cattle and other animals, animal health, handling of animal manure, type and size of crop production, provider and use of medication on animals and humans, and pesticides on crops)
- Participants (e.g., age, gender, education, marital status, and household size).
- Description of the daily work tasks.
- *Description of tools or equipment used on the farm*.
- Perceived health status (e.g., describe what you think is good/poor health for you. How would you describe your own and your family’s health? Do you experience some of the following symptoms: fever and chills, muscle and joint pain, chronic fatigue, headache, nausea, chest pain, diarrhea, vomiting, coughing or breathing problems, skin itching, and how often?)
- *Occurrence of injuries (e.g., Have you or someone in your family been injured when farming? What happened? Kind of injury?* (fracture, wound, bite, kick, crushing, burn, toxic or corrosive substance, etc.), *Injured body part*? (face, eyes, neck, back, arms/legs, fingers, chest, stomach, internal/external injuries), *Did the injury require medical treatment? Do you still suffer from the injury?)*

The interview guide also contained questions about the participants’ perception, attitude, and awareness of:

- Hazardous, physically and mentally demanding work tasks and situations (e.g., *Do you think there are risks related to your health and safety as a dairy farmer and how would you describe these risks?*)
- Hazardous farm chemicals and drugs (*Which type of farm chemicals and drugs do you use on the dairy farm? How and when do you use them? What do you do to protect yourself when you use chemicals and drugs?*).
- How to avoid getting sick or injured when farming.
- Possible benefits of a healthy and safe farm environment.
- Availability and demand for information and practical training in human health and safety when farming (e.g., *Have you received information or training regarding agricultural health and safety? Would you like information or training about this? In what form and what should the information or training contain? How could dairy farming be made more healthy and safe for you and your family in the future?)*

All interviews and transect walks were conducted on the dairy farms except for one interview, which was conducted at the university (the veterinarian). The individual interviews lasted for about 2 h, followed by 1–2 h of transect walks on the dairy farm. The transect walks took in the farm premises, the animal and machine sheds, and the pastures/crops. The interviews were held by the research team. Three of the interviews were performed in English and four interviews were translated into the local language of the Ankole tribe (Runyankole) and back-translated to English by the Ugandan colleague. To support the researchers’ notes and with the agreement of the participants, the interviews and transect walks were documented by tape recording and photographing.

### Data Analysis

Content analysis of the data collected was chosen as a qualitative validated phenomenological method (31, 32, 38). The collected material from the interviews and transect walks was anonymized and transcribed. After transcription, the text was carefully and repeatedly read to gain familiarity with the content, and all information related to the questions in the interview guide was marked, coded, and summarized at individual level. Reflections concerning the following issues were considered in the texts: What did the text contain? What did the participants say? What was important for the participants? How should the experiences and statements of the participants be interpreted? The individual texts were then analyzed and themes relating to the main issues raised by the participants were identified. These themes were summarized, and statements that described the participants’ responses were formulated according to qualitative research procedures (31, 32, 38). Transcription, analysis, and compilation of results were carried out by both authors of this paper and are presented and discussed in the following sections.

## Results

### Description of the Dairy Farms and Participants

**Figure 1:**
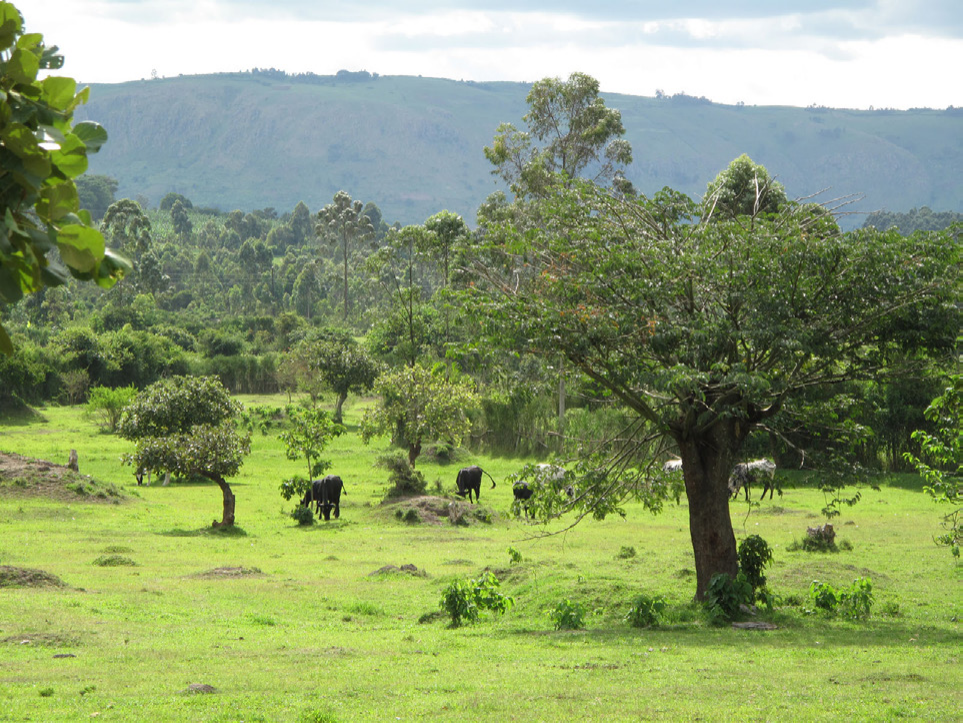
**Agro-pastoral farming in Western Uganda**. Copyright © Christina Lunner-Kolstrup.

The study comprised in total four dairy farms and interviews with six farmers and one veterinarian. Three of the four farms and farmers visited in the Mbarara District in Uganda were characterized by:

- Practising smallholder agro-pastoral farming (Figure 1) (considered here as subsistence farms with no produce surplus for market sale).
- Average herd size of 7–13 dairy cows and heifers of the traditional local Ankole breed or crossbreeds with Holstein Friesian and Ayrshire.
- 10 goats and chickens.
- Three to five pigs and sheep.
- Besides pasture for the animals, the farms grew plantain (cooking banana), sweet potatoes, beans, cassava, yams, millet, sorghum, and groundnuts on a few hectares.
- Hand tools, such as machetes, long sticks (to cut banana leaves), shovels, and hoes, were the only equipment used to cultivate the land.
- Owned and managed by male farmers.
- Little or no formal education.
- 40–70 years of age.
- Wives and children were active in farming.

The fourth farm was a large farm in a Ugandan perspective, consisting of three smaller farms, and was characterized by:

- Crop, timber, and dairy production and breeding as the respective production focus on the farms.
- 30 dairy cows and in total 125 cattle of Holstein Friesian, Jersey, and Ayrshire breed.
- 25 goats and sheep for meat and for protection of the dairy cows (potential predators choose smaller animals over larger).
- Milk yield of about 400 l of milk per day, some of which was used for household consumption, but the majority of which was sold in the market.
- 80 ha in size and included pasture, Napier grass (silage) for animal feed, timber production for building maintenance, plantain and the traditional vegetables abovementioned for human consumption.
- The land was cultivated using traditional hand tools.
- This dairy farm had a milking machine and a cooler (although they were not working because of lack of spare parts).
- A biogas unit for electricity production and a hydropower for water supply for both farming and household.
- Owned and managed by a female widow in her 60s and her 25 employees.
- Before retirement, this female farmer had worked for local government and was well educated.

### Daily Work Tasks

In Uganda, females are usually responsible for household chores and children, working in the plantation and managing smaller livestock, such as pigs, sheep, goats, and poultry. Males are often responsible for the cattle, milking dairy cows, participating in plantation work if needed, and in some cases having off-farm jobs.

A usual working day on the dairy farms visited often started early in the morning at 6 a.m. with prayer, a bath, the males milking the dairy cows, and the females feeding and watering the animals. After breakfast, work was done in the plantation and vegetable garden and the wife and daughters prepared lunch (on the large dairy farm, a young female was employed as a cook and also took care of the poultry). After lunch and rest for a few hours, the afternoon was spent in the plantation, vegetable garden, on tailoring, maintenance, household chores and cooking, and milking, feeding, and watering the animals before dinner at 6 p.m. The day ended with socializing with family and neighbors, prayer, and sleeping at 9 p.m.

### Hazardous and Demanding Work Tasks and Situation

Knowledge and awareness of health and safety risks associated with dairy farming and agriculture and prevention of injuries and diseases when farming were very low among all interviewees except for the female dairy farmer and the veterinarian. The dairy farmers, workers, and family members reported few complaints, injuries, or diseases related to dairy farming and agriculture in general. However, it was obvious from the dairy farmers’ responses that health and safety concerns, e.g., diarrhea, cough, fever, cuts while using machetes in the plantation, bruises when handling the animals, and symptoms of poisoning from using insecticides on the animals, were normal conditions, not worth talking about and considered an occupational hazard in farming. The female dairy farmer and the veterinarian explained that Ugandan farmers consider life in itself to be hard (work) and that the mental pressure and concerns regarding drought, not getting enough food for the animals and the family, having to pay for expensive medication in the event of illness and school fees for the children are more significant than a few cuts, bruises, and diseases.

**Figure 2:**
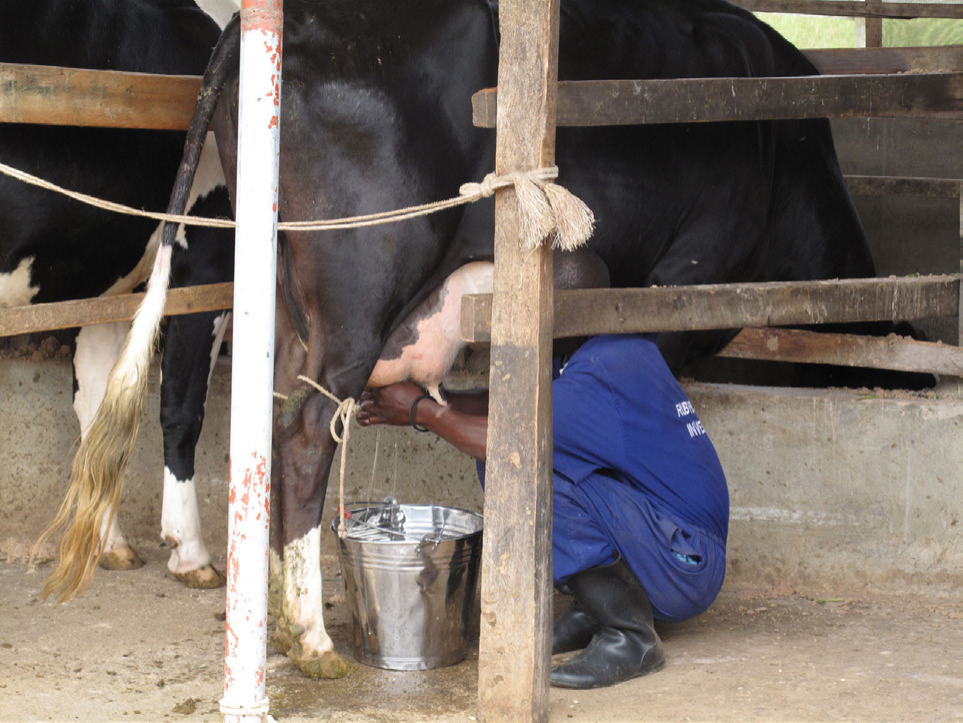
**Hand milking involving squatting and kneeling**. Copyright © Christina Lunner-Kolstrup.

However, during the interviews and transect walks, the participants highlighted several issues: hand milking the dairy cows involved squatting and kneeling, carrying the backpack sprayer with insecticide for spraying the animals, and working in the plantation were considered physically demanding and sometimes hazardous work tasks (Figure 2).

“Milking the cows is hard and my back hurts. I know I can’t milk anymore when I get old; but then I will have my children and grandchildren doing the work (Dairy farmer)”

Dairy farmers often opt to tie the hind legs of dairy cows with a rope during milking, as a safety precaution to prevent them kicking the milker (Figure 2).

“The flies are a nuisance to us and the cows and when we milk they get irritated. Milking in a shed with the cow tied would be safer than milking in the field, but we can’t afford it (Dairy farmer)”

**Figure 3:**
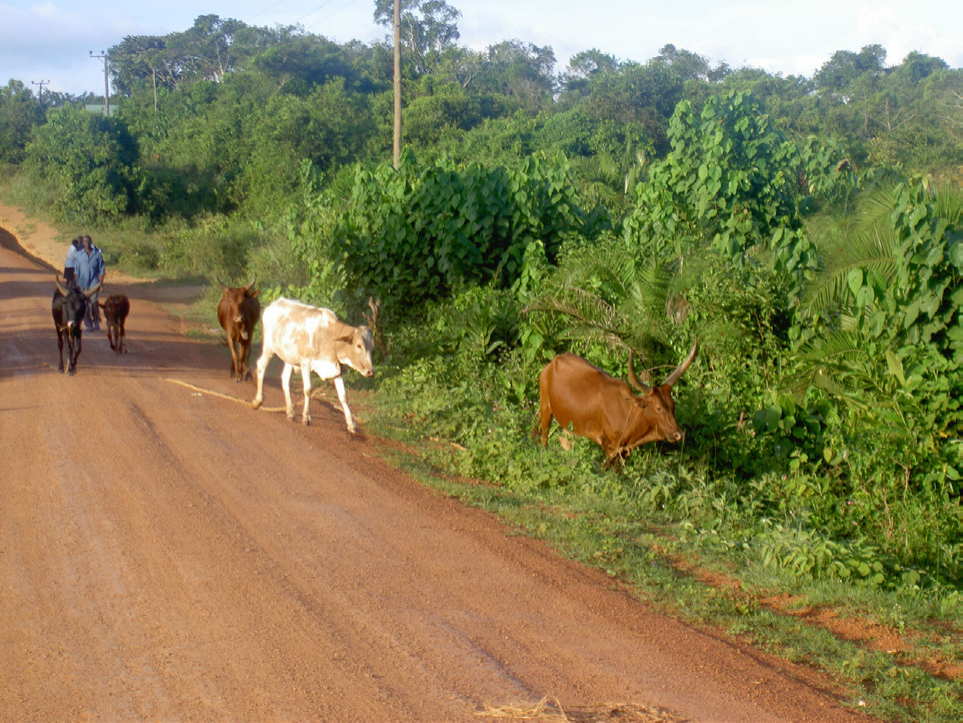
**Farmers bringing their local long-horned Ankole cows to pasture**. Copyright © Christina Lunner-Kolstrup.

Farmers in Western Uganda are traditional herdsmen, living closely with their animals for years, and have good knowledge about animal behavior. However, animals and animal handling were mentioned as possible risk factors, especially in situations when the animals are restrained, giving birth, or being moved (Figure 3). Deworming or other treatment that involved restraining animals was considered as a hazardous and physically demanding situation, as no restraining facilities were available, only human labor.

“Few farmers put up crushes for just handling the animals. It is a risky task treating unknown and semi-domesticated animals. But farmers are not to blame; we have not taught and trained them (farmers). The farmers’ don’t have much labour, so they call upon neighbours or pay hired workers to help handling the animals (Veterinarian)”

**Figure 4:**
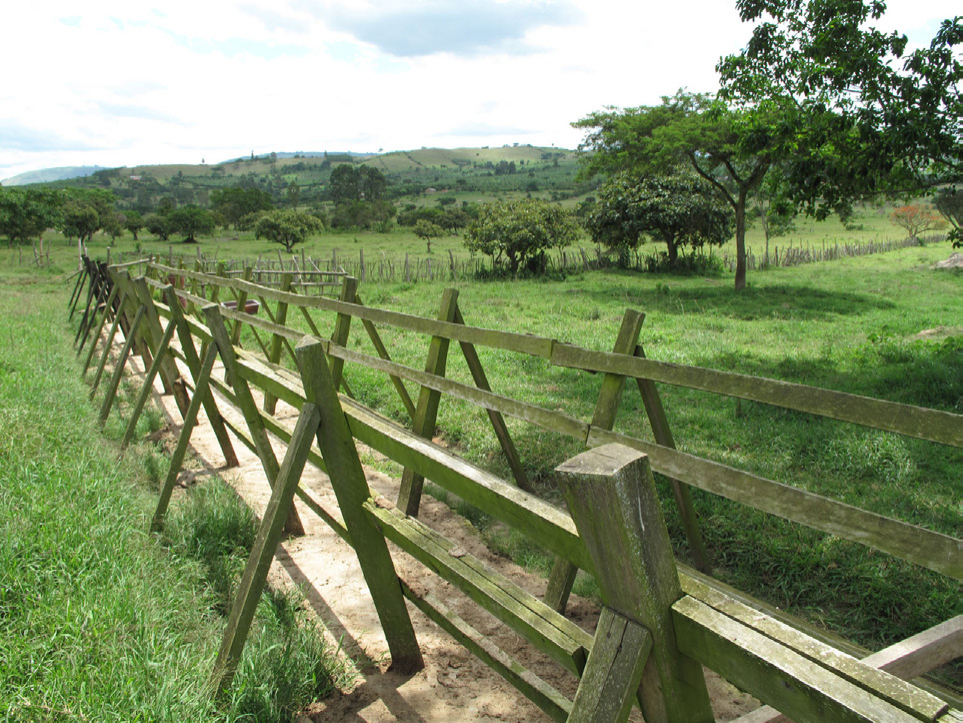
**The chute where livestock are sprayed once a week with insecticide to prevent nuisance insects**. Copyright © Christina Lunner-Kolstrup.

The most important topic mentioned by the interviewees was the use of chemicals and drugs on livestock. Once a week, the farmers gathered the animals and drove them through a chute (Figure 4). They sprayed them with insecticide, using a backpack or hand sprayer, to prevent ticks, lice, tsetse flies, and other biting nuisance flies and infections caused by these insects [such as East Coast Fever, Bovine Babesiosis (also called Redwater or Tick Fever) and Anaplasmosis (also called Gall Sickness)]. The insecticide used for spraying the animals was bought in the local veterinary drugstore, but this store was seldom run by a veterinarian. The regulations on providing chemicals and drugs for both humans and animals have been delegated to the private sector by the Ugandan government and no prior education or training is required for selling drugs or opening a drug store.

“One I know was working in the service commission and when he left, he went into dealing agrochemicals. But he didn’t have any prior training in dealing drugs; the law is not embracing, the policies are there but not implemented – it is lacking and the public service doesn’t regulate the private sector (Dairy farmer)”

The storekeeper often has limited or no knowledge and gives no information to the farmers about the chemicals or drugs except for dosage. Furthermore, the labels on medicine packaging were small and the farmers interviewed did not understand the text or relate to the warning signs given on the labels.

“They give you simple instructions on how to mix and how to apply – but not how to protect yourself (Dairy farmer, translated)”

Another critical problem identified was that several farmers in the region were illiterate and would have needed visual information or practical training and instructions. Spraying was conducted without the use of PPE such as face masks, overalls (except for the large dairy farm), eye goggles, gloves, or rubber boots. Those interviewees who were aware of the existence of PPE considered it too difficult to use, too expensive, and difficult to obtain. The farmers explained that they usually felt unwell, dizzy, vomited, and had pain and a burning feeling in the face and eyes after spraying their animals. The symptoms of poisoning lasted from a couple of hours to several days. The farmers used indigenous medical herbs, showered, or rested for some hours until the symptoms had disappeared. However, the symptoms could be so severe that they needed treatment and bought medication at the local drugstore without a medical prescription or seeing a medical doctor.

In order to maintain good health and avoid getting sick, the participants stated the importance of eating well and relying on local indigenous food (meaning without pesticides). They seldom fell sick and if they did, it was just local diseases and fever. Fever, coughing, and diarrhea are common among Ugandan farmers and are often related to malaria, tuberculosis, Salmonella, Q-fever, leptospirosis, or brucellosis (zoonotic disease where humans are infected through, e.g., consuming unpasteurized milk). Almost all the interviewees were unaware that some diseases could be transmitted from animal to human and vice versa; they did not know of brucellosis, typhoid, or Salmonella, just diarrhea, fever, or a simple cough.

The dairy farmers seldom visited the medical clinic (too expensive, no trust in medical experts, and too far away). If they had a fever, they sometimes bought medicine in the local drug store. This raised the sensitive topic of farmers using animal medicine for human treatment.

“Here I am (as the farmer), I’ve been growing up with this animal, it falls sick, it gets a fever (we call it fever), it’s given medicine and it heals – so, I have a fever, I can share the drug. They just reduce the dose! So many mills – 5 mills (millilitre) for an animal and 2 mills for a human! (The veterinarian explained the reasoning among farmers)”

### Benefits of a Healthy and Safe Farm Environment

The female dairy farmer was a progressive farmer and viewed her farm as a business. She stated that a healthy and safe environment for animals and humans would result in profitable production and healthy and happy workers. She had developed routines for milking, hygiene, animal handling, feeding, animal book keeping, use of chemicals, and human safety. The female dairy farmer provided training for her employees and other neighboring farmers on how to deworm the cattle and information concerning the time restriction for using meat and milk after treatment. She viewed employees as a resource and had a great interest in employee management and how to recruit, train, and retain skilled farm workers. She took good care of her employees; she trained them how to manage dairy cows and keep records, gave them fair wages, housing conditions, and access to medical care, and believed that managing the human capital on her farm is very important and necessary for her survival as a farmer. In addition, she chose to employ labor instead of investing in technical equipment.

“I’m not really keen on mechanising because we have the human resource everywhere and I could just as well employ as many (workers) as possible, so that they also can earn their livelihood from here (Dairy farmer)”

### Availability and Demand for Health and Safety Education

Information and practical training on agricultural health and safety in the region were non-existent. Almost all interviewees were eager to gain knowledge and attend training on how to identify and handle risks in order to prevent diseases and injuries when working in the fields and with livestock. The farmers had confidence in non-government organizations (NGOs) and veterinarians and preferred them, in collaboration with agricultural health and safety specialists, to hand out information and conduct practical training courses. The dairy farmers also mentioned the urgent need for simple safety aid kits and PPE, such as face masks and gloves. However, one of the farmers commented on the need for practical training regarding PPE:

“You need to show them (the farmers), not just explain, the importance of using them (PPE), otherwise you will just give it to them and they will not use it. You need to show them the associated risks and dangers on the farm, show them how to use it (PPE) and then you can provide it (Dairy farmer, translated)”

## Discussion

### Level of Knowledge and Awareness

The results obtained in this study indicate that the dairy farmers interviewed had low knowledge and awareness of risk factors and health and safety issues relating to dairy farming. They experienced physically demanding and hazardous work tasks related to working with their livestock and farm work in general, which jeopardized their safety and health. These results are consistent with findings in previous research studies and reviews among farmers in Africa (21–27). Cuts and bruises, both severe and less severe, were often treated at home or by a neighbor with specific knowledge of healing herbs. The farmers did not consider these injuries worthwhile noting, reporting, or seeking medical care for. The farmers seldom visited medical clinics, probably because of low convenience, being too geographically remote, lack of access to transportation, lack of financial means to pay a medical doctor, lack of confidence in the medical services, or lack of adequate and available health care.

A systematic occupational health and safety study conducted in Gambia showed that farmers were exposed to a number of risk factors which seriously affected their health (23). This study also comprised extension workers and the results showed discrepancies regarding the comprehension and severity of injuries, indicating under-reporting among farmers. Under-reporting might also have occurred in this study, but for different reasons. Farmers and ruralists in developing countries face poverty (39) and conduct farming as the only option for obtaining their daily livelihood, and therefore they may be more prone or have no other choice than to accept hazards and injuries as part of their occupation. A number of factors, such as weather, drought, hurrying to complete work tasks in rain-fed agriculture with two short rain seasons, and uncertainty about profitable crop harvests and livestock yields, put farmers in a state of anxiety, and they may be more vulnerable to injuries and perhaps even mental strain (22).

Under-reporting is probably attributable to a low level of knowledge and awareness concerning the hazards in dairy farming and how it could affect farmers’ health and safety. Information or practical training on prevention of injuries and diseases relating to dairy farming or agriculture in general was not available to the interviewees, but earnestly requested, as the region relies heavily on crop and livestock production. Most of the farmers interviewed were illiterate, which is not uncommon in many developing countries (40), and they had learned farming practices from elder peers. It is a major challenge to provide information and practical training to increase knowledge and awareness about health and safety that is adapted to the educational level of these farmers. This challenge of developing appropriate, participatory, and practice-based courses has also been acknowledged in other studies (41, 42).

### Use of Agrochemicals on Livestock Jeopardizes Human Health and Safety

The most problematic issue identified was the use of agrochemicals and drugs in livestock production. Illiterate farmers handling dangerous agrochemicals, without proper instructions and PPE, face an increased risk of allergic or irritant skin reactions and acute and chronic intoxication. Besides directly affecting farmers’ health through dermal contact or inhalation, misuse of agrochemicals also poses a health risk in terms of milk contamination. This is a serious food safety problem for milk consumers, especially if no time restriction is applied after treatment. Several residues of acaricides and pesticides have been found in cow’s milk in developing countries (25, 43, 44). The use of agrochemicals and drugs was reported to be associated with insufficient information from the drugstore or on packaging and misuse of medication due to ignorance.

The farmers in Western Uganda are mainly pastoralists and are dependent on high milk yield as one of their main protein sources. In order to boost milk production, crossbreeding between local cattle and imported high-yielding Holstein Friesians cattle is common. These crossbreeds have lower susceptibility and resistance to local diseases and require antibiotics, anti-parasitic drugs, and intensive tick protection to survive. Without such treatment, there is not only a threat to animal health and a risk of heavy losses of livestock but also a threat to human health (25). Consequently, comparative studies have shown that cattle crossbreeds in African countries are treated with acaricides (pesticides that exterminate members of the arachnid subclass Acari, which includes ticks and mites) up to twice a week and the user often employs no form of quality control or restriction (25).

The farmers interviewed in this study used insecticides for spraying animals to protect them from insects and parasitic diseases. This was done with old inefficient sprayers, with liquid probably leaking and dropping on the farmers’ skin through soaking clothes. Studies have shown that overdosing is erroneously believed by farmers to enhance the effect, but instead it increases the risk of exposure and poisoning because of misuse (26). The farmers interviewed did not use PPE in most cases, and in general, PPE use is uncommon in the region, due to lack of availability, comfort, and affordability, as reported previously for other African countries (23, 26, 27, 45, 46). The farmers interviewed here reported body symptoms of pesticide poisoning with a duration which varied from hours to several days. Unfortunately, a number of the agrochemicals available in many developing countries are banned, unregistered, outdated, and unlabeled pesticides sold uncontrolled and without restrictions at local markets or small shops by illiterate or ignorant vendors (25–27). Lack of legislation and enforcement regarding PPE and sale of agrochemicals has also been demonstrated in other studies (23, 27). According to the interviewees and studies in other developing countries, fake, substandard, and diluted drugs are common (25). The pesticides used by the farmers in this study were labeled but, unlike in a similar study conducted among farmers in Gambia (27), the farmers in this study did not understand or relate to the symbols and warning signs on the packaging. This means that information and instructions need to be adapted to users and their language and culture.

### Animal Drugs Used for Human Treatment

The veterinarian interviewed raised the issue of use of animal drugs for human treatment. This was not mentioned by the farmers, which according to the veterinarian and the Ugandan coauthor could be because the farmers could not distinguish between human or animal drugs, or because of taboo and shame. Use of animal drugs for human purposes has been reported previously in a study among Gambian farmers (27), where 81% knew of farmers and field workers using pesticides for non-agricultural purposes (27).

### Zoonotic Diseases a Serious Health Risk

Cultural and religious beliefs may play an important role concerning zoonotic diseases. The farmers interviewed were unaware of zoonotic diseases and found it difficult or impossible to imagine or comprehend that they could get diseases from their animals. In a study among Gambian farmers, headache (35%) and chronic cough (21%) were frequently reported, and, as in this study, awareness of zoonotic diseases and other diseases relating to agriculture was absent (23).

East Africa has a high zoonotic burden (25, 28) and infectious diseases relating to agriculture are playing an increasing role (28, 47). In developed countries, 20% of human illness and fatalities are attributable to zoonotic diseases and one can only imagine the scope and severity in developing countries (28). Several studies have shown that zoonotic diseases are a key concern in developing countries and show a strong association with poverty, hunger, and livestock production (28). Furthermore, the rural population, including farmers, in developing countries is vulnerable, as inadequate diet and exposure to endemic and occupational diseases, in combination with poor sanitation, inadequate housing, malnutrition, and various parasitic and bacterial infections, have been shown to constitute a vast risk concerning health (39). A possible intervention in order to prevent infections and zoonotic diseases and to improve the health status could be vaccination of the farm population. Availability and offering chemoprophylactic medication could also be an option, but could carry an associated risk of medical resistance, e.g., to antibiotics and anti-malarials.

### Strategies for Health and Safety Improvements

Agriculture, a major driver in Ugandan economy (48), could be expected to generate government interest and concern for the health and safety of its producers (farmers, agricultural workers, and their families). Investment in occupational health and safety would add value to the country by resulting in improved working conditions, higher labor productivity, and a healthier farm population. One way to increase awareness and knowledge could be by comprehensive campaigns in rural areas providing educational and illustrative information and participatory practical training courses in the local language. These measures need to be implemented in interdisciplinary and participatory collaboration between NGOs, veterinarians, medical doctors, farmers, and role models (like the well-educated female farmer interviewed here). More importantly, farmers must trust their educators, and training must be performed with respect to the cultural and religious beliefs and norms of the region.

In Mali, special field schools in a community of cotton growers trained farmers in alternative methods of pest control and succeeded in nearly eliminating the use of toxic pesticides (42). In Gambia, researchers found that a community-based participatory approach and cultural acceptance were essential for successful implementation of interventions to improve health, safety, and productivity among smallholder female farmers (41).

Furthermore, simple PPE solutions should be introduced, such as long sleeves and trousers, boots, gloves, and facial masks, and information concerning personal hygiene (washing clothes and showering after pesticide use) when applying agrochemicals. Moreover, enforcement, monitoring, inspection, and education of vendors of agrochemicals and medical drugs should be prioritized and implemented in order to reduce uncontrolled sales by unknowledgeable vendors.

### Study Limitations

The study comprised a small sample of Ugandan farmers and farm workers (six interviewees on four farms and one interview with a veterinarian) in a region with high livestock density. In order to find farmers who would agree to be interviewed and willing to share experiences, we chose sample selection by convenience using local contacts to identify dairy farmers in the region. Based on the limited data material, we cannot claim that the results are representative for all Ugandan dairy farmers. However, the intention was not to generalize, but to explore and highlight important occupational health and safety issues for individual dairy farmers. The farmers mentioned many of the same issues and the last interview did not bring new information to the material, meaning that saturation had been reached. Furthermore, our main findings are supported by other occupational health and safety studies conducted in Africa, which also contributes to the credibility of the study.

Culture and language barriers can be a limitation, but this study was a cross-country collaboration, which was a strength. Both authors were present at all interviews and the university colleague from Uganda Martyrs University, who specializes in agriculture and is familiar with the local culture and language, performed the interviews in the local language. Furthermore, interpretation of the collected material and discussion of results were performed by both authors, in order to reduce the bias of cultural and language barriers. Over- or under-reporting of incidents could have affected the results. Lack of awareness and knowledge of occupational health and safety indicates the likelihood of under-reporting, and thus the topic is of immense importance to address. Several of the farmers were illiterate and, therefore, interviews were chosen as a suitable method. The use of interviews also provided the possibility for explaining and asking sub-questions. The farmers interviewed sometimes had difficulties understanding the health and safety concepts, but as the Ugandan colleague is familiar with the field of occupational health and safety, the culture and the local language, he was able to explain matters to the farmers. The use of interviews as a method limited the generalizability of the findings, but increased the possibility of obtaining a rich picture and a more profound understanding of the issues.

## Conclusion

Studies that can lead to improved human health, safety, sustainable development, poverty reduction, a fair livelihood for farm populations, and gender equality in low income countries can provide various benefits for individuals and for the community and the country at large. The results obtained in this study indicate that the level of knowledge and awareness of agricultural health and safety risks, disease, and injury prevention among the Ugandan dairy farmers interviewed was low. The farmers mentioned few agriculture-related complaints, injuries, or diseases except poisoning from using agrochemicals. Training on health and safety in agriculture is urgently needed in the region of the farmers interviewed. The study also highlights some of the key issues to be addressed in future research such as the zoonotic burden, the use of animal drugs for human treatment, limited use of PPE, education of agrochemical strategies retailers, and the need for participatory approaches for successful implementation of health and safety prevention. This study comprised few dairy farmers and makes generalization not possible. However, the results are supported by other research studies implying that the findings in this study most likely mirror the situation among farmers in other developing countries.

## Author Contributions

CK developed the research idea, applied for funding, designed the project, performed the literature review, collected and analyzed data, and wrote most of the manuscript. TS participated in planning the interviews, selected the interviewees, translated from the local language to English, and participated in interpreting the results and writing the manuscript. Both authors revised the manuscript and approved the final draft.

## Conflict of Interest Statement

The authors declare that the research was conducted in the absence of any commercial or financial relationships that could be construed as a potential conflict of interest.
